# 99. Predictive model for serious bacterial infections in infants less than 90 days of age based on clinical data and a combination of high-threshold inflammatory biomarkers

**DOI:** 10.1093/ofid/ofad500.015

**Published:** 2023-11-27

**Authors:** Halima Dabaja-Younis, Nadeen Makhoul, Rozeen Abu Shqara, Ranaa Damouni Shalabi, Anat Reiner-Benaim, Manfred S Green Green, Imad Kassis

**Affiliations:** Rambam Health Care Campus, Haifa, Hefa, Israel; Rambam Health Care Campus, Haifa, Hefa, Israel; Rambam Health Care Campus, Haifa, Hefa, Israel; Rambam Health Care Campus, Haifa, Hefa, Israel; University of the Negev, Beer Sheva, HaDarom, Israel; University of Haifa, Haifa, Hefa, Israel; Rambam Health Care Campus, Haifa, Hefa, Israel

## Abstract

**Background:**

Serious bacterial infection (SBI) is a life-threatening condition affecting infants less than 90 days of age, and prompt differentiation from non-bacterial infection (NBI) is crucial. Recent guidelines recommend a combination of low-threshold inflammatory biomarkers that need further validation. The aim of this study is to develop predictive models for SBI in infants < 29 days and in infants aged 29-90 days. The study uses detailed clinical data and inflammatory biomarkers with high thresholds.

**Methods:**

This retrospective cohort study was conducted between 2010 and 2019 in a tertiary referral hospital in northern Israel. The study included previously healthy and term infants < 90 days of age who were admitted to the pediatric units of the hospital with febrile illnesses. SBI was defined as bacteremia, meningitis, or urinary tract infection, while NBI was defined as absence of signs of bacterial infection. Detailed clinical and laboratory data were collected for each of the two age groups.

Chart flow of infants included in the study

**Figure:**
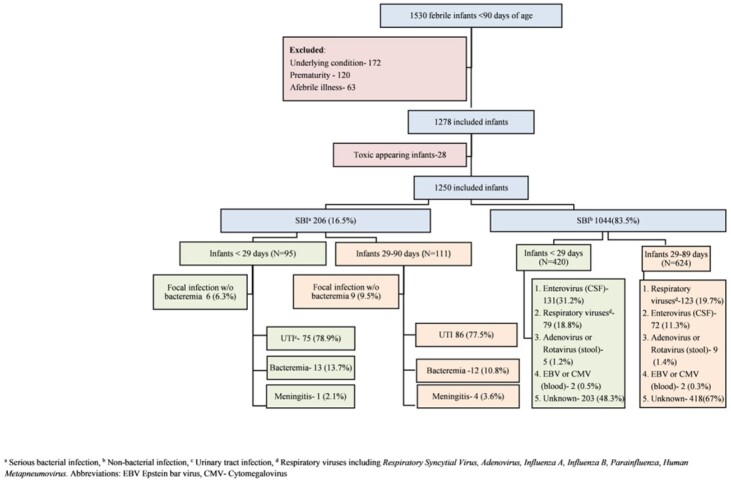

a Serious bacterial infection, b Non-bacterial infection, c Urinary tract infection, d Respiratory viruses including Respiratory Syncytial Virus, Adenovirus, Influenza A, Influenza B, Parainfluenza, Human Metapneumovirus. Abbreviations: EBV Epstein bar virus, CMV- Cytomegalovirus

**Results:**

A total of 1250 infants met the inclusion criteria, of whom 515 (41.2%) were < 29 days old and 735 (58.8%) were between 29 and 89 days old. In the younger group, 95 (18.4%) had SBI. Risk factors for SBI were diarrhea, absolute neutrophil count (ANC) > 7,500mm^3^ and CRP > 3mg/dL. Viral symptoms in family members proved to be protective. In the older group, 111 (15.1%) had SBI. Risk factors for SBI were ANC > 7,500mm^3^ and CRP > 3mg/dL, while male sex, viral symptoms in family members and diarrhea were protective. The study developed a predictive model, the "NeoSBIscore", based on the above predictors, focal infection on physical examination or abnormal urinalysis. The model showed a sensitivity of 94.7% and a negative predictive value (NPV) of 96.9% in infants younger than 29 days and a sensitivity of 93.7% and a NPV of 96.2% in the older group. Only cases with urinary tract infections or bacteriuria were missed in both age groups.

"NeoSBIscore" model to identify infants admitted with febrile illness and at increased risk for serious bacterial infection.

**Figure:**
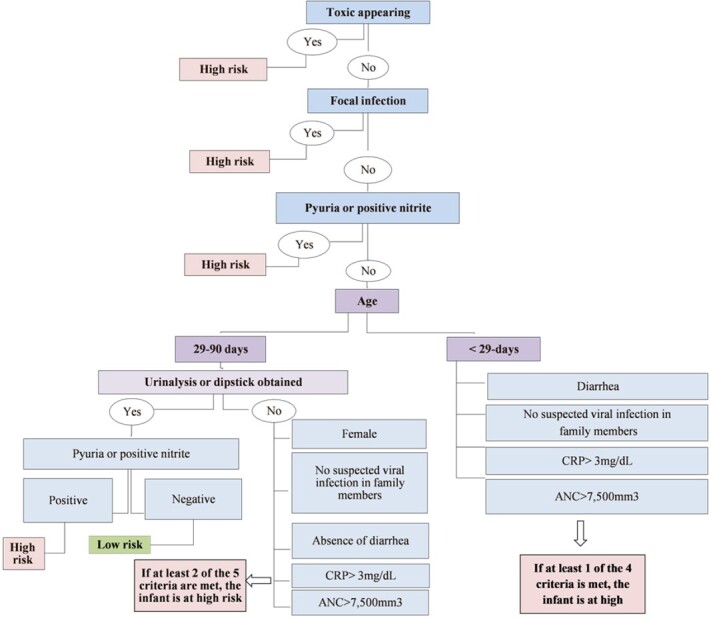

**Conclusion:**

The "NeoSBIscore" model successfully identified infants with SBI, and the use of higher-threshold inflammatory biomarkers did not result in missed cases of SBI, with the exception of a few cases with UTI or bacteriuria. However, external validation is required before this model can be used in clinical practice.

**Disclosures:**

**All Authors**: No reported disclosures

